# Detection of microcirculatory impairment by transcutaneous oxymetry monitoring during hemodialysis: an observational study

**DOI:** 10.1186/1471-2369-15-4

**Published:** 2014-01-08

**Authors:** Ygal Benhamou, Loic Begarin, Nathalie David, Nicole Cailleux, Catherine Bessin, Herve Lévesque, Stephane Edet

**Affiliations:** 1 Department of Internal Medicine, Rouen University Hospital, 1 Rue de Germont, 76031 Rouen, France; 2 Department of Vascular Surgery, Rouen University Hospital, Rouen, France; 3 Department of Nephrology, Dieppe General Hospital, Dieppe, France

**Keywords:** Peripheral arterial disease, Critical ischemia, Transcutaneous oxymetry, Hemodialysis

## Abstract

**Background:**

Little is known about the effects of intermittent hemodialysis on microcirculatory perfusion. The aim of this study is to assess the effects of hemodialysis on microvascular perfusion using transcutaneous oxymetry (TCPO2).

**Methods:**

In this observational study, hourly TCPO2 measurements were performed during hemodialysis sessions. Ankle brachial index (ABI) was carried out to classify patients according their vascular condition.

**Results:**

50 patients (mean age 70 ± 8 years old) were enrolled. Mean TCPO2 decreased significantly on average 23.9% between start and finish of hemodialysis. Severe ischemia (TCPO2 < 30 mmHg) and critical ischemia (TCPO2 < 10 mmHg) occurred during dialysis in 47.1% and 15.5% respectively. Critical ischemia occurred only in limbs with ABI < 0.9 (8.3%) or > 1.3 (28%). Patients with critical ischemia experienced a significantly larger decline in mean blood pressure (32.4 ± 26.1 mmHg vs 12.7 ± 10.7 mmHg; P = 0.007) and a more pronounced ultrafiltration (45.55 ± 16.9 ml/kg vs 35.17 ± 18.2 ml/kg; P = 0.04) compared to patients without ischemia. Clinical outcomes (death or vascular procedures) were five times more frequent in patients who had developed critical ischemia (55.7% vs 10.1% P = 0.01). The elevated age of patients, the low basal value of TCPO2, and the occurrence of critical ischemia were more frequently associated with clinical outcome (*P* = 0.03, *P* = 0.048, *P* = 0.01 respectively).

**Conclusions:**

This study demonstrates that hemodialysis induces microcirculatory injury, dependent on blood pressure reduction, peripheral vascular state and ultrafiltration. The occurrence of critical ischemia is associated to pejorative patient outcome and therefore, TCPO2 seems to be useful to avoid potential distal tissue damage during hemodialysis.

## Background

Peripheral arterial disease (PAD), traditionally defined by an ankle brachial index (ABI) of ≤ 0.9 is highly prevalent in dialysis patients and considered as a marker of poor prognosis [1-4]. Patients with mediacalcosis (ABI > 1.3) are also considered as presenting high vascular risk [4]. Short intermittent hemodialysis (HD) exerts significant hemodynamic and metabolic effects. Conventional HD is known to be associated with endothelial injury, elevated levels of oxidative stress and an inflammatory state leading to accelerated atherosclerosis [5-7]. Patients undergoing hemodialysis, have more frequent PAD procedures (lower extremity amputations and bypasses), compared to the general population, with poor prognosis [8]. Thus, early diagnosis of PAD and subsequent optimized vascular perfusion in HD patients deserves special attention. Silent limb microcirculatory injury is probably underestimated and should be recognized to prevent the onset of macrovascular limbs complications. Therefore, improvement of the hemodynamic tolerability of HD on microcirculatory vessels is one way of reducing tissue damage. However, despite improvements in HD techniques, unevaluated episodes of distal ischemia-reperfusion during HD, remains high and could partially explain the high incidence of vascular complications.

The use of transcutaneous oxymetry (TCPO2), a non invasive vascular tool, allows monitoring of local arterial blood flow and skin oxygenation [9,10]. TCPO2 is influenced by both central arterial oxygen content and local factors, such as tissue perfusion. The positive role of TCPO2 in quantifying the degree of ischemia is well known in the population at large [11]. Values of TCPO2 in a decubitus position less than 30 mmHg or less than 10 mmHg are considered as either severe or critical ischemia respectively [11]. However, TCPO2 has been poorly studied in hemodialysis and its performance in detecting severe ischemia in hemodialysis patients is not well known. Sato et al. have shown that mean TCPO2 value was significantly lower in HD patients without PAD than in control subjects [12].

Our group has recently reported the usefulness of TCPO2 in improving PAD diagnosis and assessing its severity in hemodialysis populations. We have shown that a TCPO2 lower than 40 mmHg at onset of hemodialysis might identify patients either at high risk of death or requiring vascular treatment [13].

However, only few data on the monitoring of cutaneous microcirculation using TCPO2 during hemodialysis sessions are available. Santesson et al. investigated the effects of three consecutive hemodialysis sessions on peripheral microcirculation and macrocirculation and have shown that TCPO2 decreased significantly during hemodialysis, regardless of blood pressure reduction [14]. Unfortunately, this latter study took into account, neither the hemodialysis parameters (plasmatic volume reduction, total ultrafiltration) nor the peripheral vascular conditions of the patients. The aims of this study are firstly to clearly state the hemodynamic effects of HD on microcirculatory perfusion using TCPO2 as a monitoring tool and secondly to recognize some parameters leading to pejorative outcomes (cardiovascular death or vascular treatment requirement).

## Methods

This observational study was approved by the Ethics Committee of Rouen University Hospital, and all patients gave their informed consent prior to inclusion.

### Patients

Patients were recruited from a single hemodialysis center, between April and September 2010, and followed-up prospectively for one year. The study population consisted of patients with ESRD undergoing maintenance hemodialysis. Inclusion criteria were: patients treated with maintenance hemodialysis three times per week since at least 3 months and a session length of 240 minutes. Patients with an acute renal failure, or with a recent cardiovascular event (< 1 month) ie stroke, acute limb ischemia, acute coronary syndrome were excluded. Of the 50 patients included for analysis, 39 (78%) received dialysis with arterio-venous fistulas, while the others patients had central venous catheters. The monitor of HD was Integra® (Hospal, Mirandola, Italy) and only synthetic high-flux membranes were used. The dialysate flow was 500 ml/mn (n = 42) or 750 ml/mn (n = 8) while the blood flow ranged from 300 ml/mn (n = 13) to 450 ml/mn. Relative blood volume (RBV) was calculated during the session, expressed as a percentage of initial blood volume, measured continusly by Hemoscan® optical densitometer (Hospal, Mirandola, Italy).

Prior diagnosis of PAD was established based on ultrasonographic or angiographic results or, previous vascular surgical or endovascular revascularizations.

### Methods

#### Transcutaneous oxymetry (TCPO2)

All baseline TCPO2 measurements were performed at the beginning of the HD session (T0) after measurement of oxygen parameters by pulse oximetry monitoring. Hourly TCPO2 measurements were taken on both limbs until the end of the session (T240). TCPO2 was measured at the dorsum of both feet in the first intermetatarsal space with the patient n supine position, in an air-conditioned room maintained at 22°C. A double-sided adhesive ring and contact liquid supplied by the manufacturer was used to obtain a hermetic area for measurement. TCPO2 was measured as above [9,15] by an electrochemical transducer (TCM^tm^3, Radiometer GMBH, Copenhagen, Denmark). The transducer was heated to 44°C to increase the permeability of the skin to oxygen molecules at the measuring site. Calibration period was, on average, 10 minutes and the TCPO2 signal was continuously recorded on computer software during 10 minutes. A value lower than 40 mmHg is considered to be pathologic. Severe ischemia and critical limb ischemia were retained when TCPO2 measurements were lower than 30 and 10 mmHg respectively [4,16]. We calculated the variation of TCPO2 corresponding to the variation of TCPO2 between start and finish of hemodialysis.

#### Ankle brachial index (ABI) measurement

ABI was calculated as the ratio between the higher value of systolic blood pressures at the anterior or posterior tibial artery for each leg and, the brachial systolic pressure recorded. Brachial systolic pressure was measured at the shunt (vascular access) free arm. Systolic pressure was detected with a hand-held 8 –MHz Doppler probe while the patient was supine after 5 minutes rest. Only ABI values of 0.90 or less were considered as abnormal or pathologic and indicators for PAD diagnosis [16]. ABI values of 1.3 or greater or incompressible arteries at ankle level were considered to indicate mediacalcosis [17]. ABI values from 0.9 to 1.3 were considered as normal. ABI was determined by the same operator during the entire study.

### Follow-up

Follow-up visits were scheduled at 12 months from study inclusion. During follow-up, patients were asked about the necessity of a vascular treatment (revascularization or amputation). Death from any cause was noted. A combined end point (pejorative outcome) was then defined including death and vascular procedures requirement.

### Statistical analysis

Demographic data and clinical features were analysed using descriptive methods. Quantitative variables were expressed as mean +/- standard deviation and compared using the Student-t-test, or Mann–Whitney U test as appropriate. Comparison between groups was performed using Chi-square test and in the 2*2 tables, Fisher’s exact test as appropriate. The independence of each limb was confirmed by intra-class correlation coefficient measurement.

Statistical analyses were performed using STATVIEW (Version 5.0 de SAS Institute Inc.) program. P value lower than 0.05 was considered statistically significant.

## Results

### Baseline patient characteristics

The study included 50 patients from April to September 2010. We distinguished four groups of renal disease responsible for ESRD: diabetic nephropathy (16%), vascular nephropathy (22%), chronic glomerulonephritis (10%) and others (52%) such as chronic interstitial nephritis, polycystic kidney disease and nephrectomy.

Patients clinical features are listed in Table 1. The patient population was 27 males (54%) and 23 females (46%). Mean age and body mass index were respectively 70 ± 8 years old and 26.9 +/- 6.6 kg/m^2^. Hypertension (92%), diabetes (32%) and dyslipidemia (76%) were the most prevalent comorbid chronic conditions. Approximately two thirds of patients were known to have at least one cardiovascular injury (stroke, transient ischemic attack, myocardial infarction). Fifteen patients (30%) suffered from PAD (intermittent claudication for 12 of them and rest pain for 3 of them). Two patients had a previous angioplasty of the superficial femoral artery and one had been amputated.

**Table 1:** Baseline characteristics of patients

**Demographic and clinical characteristics of patients**	**Number**	**%**
Male/Female	27/23	54/46
Age (y)	70+/- 8	
Diabètes	16	32
Hypertension	46	92
Dyslipidemia	38	76
**Cardio Vascular History of Patients**		
Smoking patients	22	44
Past	16	32
Active	6	12
Stroke	11	22
Intermittent claudication	12	24
Rest pain	3	6
Ischemic cardiopathy	18	36
**Dialysis vintage (years)**		
> 10 years	07	14
5 years < dialysis < 10 years	11	22
< 5 years	32	64
**Vascular statue of Limbs**		
Peripheral arterial disease (ABI < 0.9)	15	15
Normal vascular state ( 0.9 < ABI < 1.3)	34	34
Mediacalcosis (ABI > 1.3)	50	51

ABI was analysed for each leg (one patient was amputated), the proportion of ABI lower than 0.9, greater than 1.3 and normal was 15% (n = 15), 51% (n = 50) and 34% (n = 34) respectively.

Most patients (64%) had received dialysis less than 5 years prior to the study. Median length of dialysis is 3.2 years [0.7-29.5].

### Baseline TCPO2, evolution and variation of TCPO2

At the beginning of the dialysis session, mean oxygen saturation value for all patients was 95 ± 6%, whereas mean baseline TCPO2 for all limbs was 45.7 ± 15.5 mmHg (n = 99). Eighty three of them had a TCPO2 greater than 30 mmHg and 14 limbs had a TCPO2 lower than 30 mmHg without critical ischemia. Among limbs with an ABI < 0.9 (n = 15) or > 1.3 (n = 50), the mean baseline TCPO2 was reduced compared to the normal ABI group (38 ± 16.9 mmHg; 46.5 ± 11.4 mmHg and 53.5 ± 15.5 mmHg respectively; P = 0.0002 and P = 0.02).

**Figure 1:**
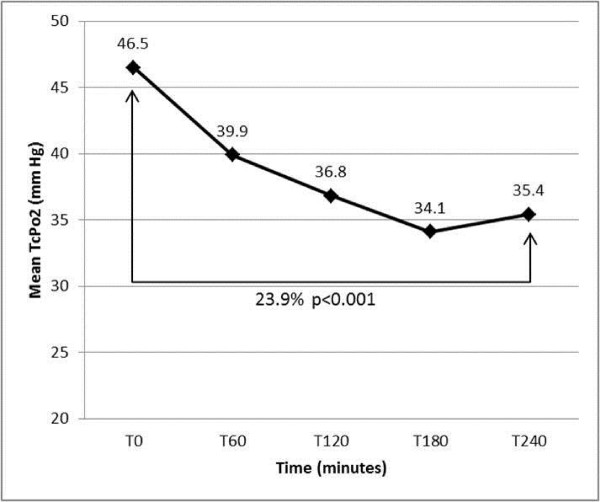
**Evolution of TcPO2 values at different times of measurement during haemodialysis.** P < 0.05 was considered statistically significant.

**Figure 2:**
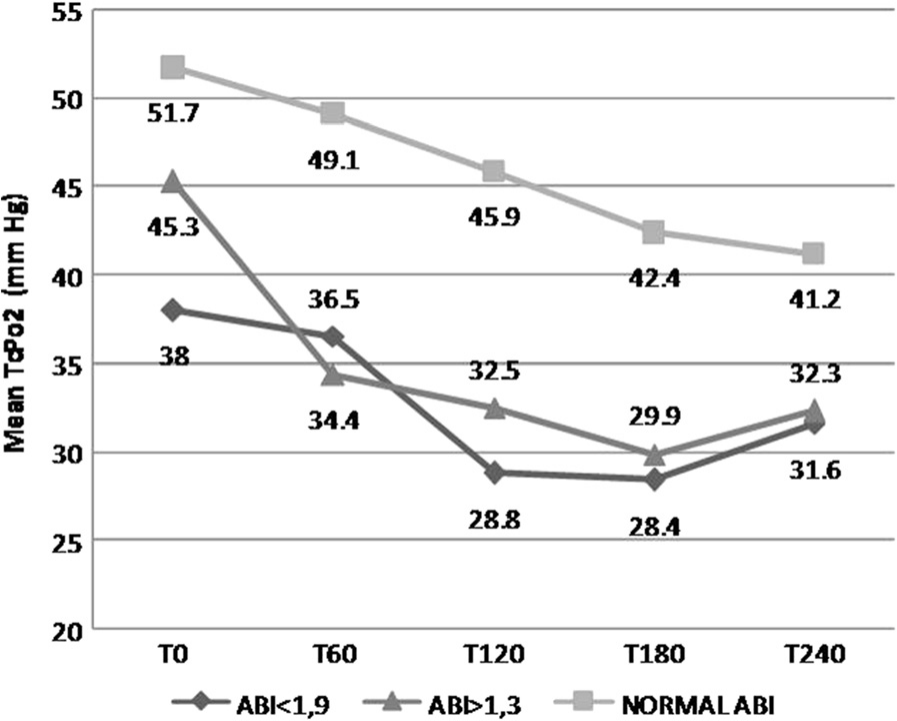
Evolution of TcPO2 values at different times of measurements during hemodialysis according to ABI Group.

In overall limbs, mean TCPO2 decreased significantly during dialysis on average by 23.9% between T0 and T240 (45.7 ± 15.5 mmHg vs 33.5 ± 15.6 mmHg; P <0.0001) (Figure 1). The absolute change of TCPO2 values was similar between groups of limbs regarding their ABI measurement (ABI < 0.9, ABI > 1.3 and normal ABI (Figure 2).

Severe ischemia (TCPO2 < 30 mmHg) and critical ischemia (TCPO2 < 10 mmHg) were always asymptomatic and occurred during dialysis in 47.1% and 15.5% of limbs respectively. Most of severe ischemia (87.5%) or critical ischemia (93.3%) occurred at the third hour of dialysis (Table 2).

**Table 2:** Evolution in the number of limbs according to their ischemic status during the hemodialysis session

	**Start of HD**	**T60**	**T120**	**T180**	**End of HD**
**Critical ischemia**	2	8	11	12	6
**Severe Ischemia**	14	22	24	26	35
**No ischemia**	83	69	64	61	58

Considering patients, at the beginning of HD session, 48 patients had TCPO2 > 10 mmHg in both limbs. During the HD session, critical ischemia and severe ischemia occurred in 9 and 24 patients respectively. Only 15 patients remained with a TCPO2 value greater than 30 mmHg.

### Predictive factors of ischemia during hemodialysis

The onset of ischemia (severe or critical) was not related to demographic characteristics such as age (67.8 ± 15.3 y vs 75.3 ± 8.7 y; P = 0.13), hypertension (88.6 ± 5.5%, vs 100 ± 0%; P = 0.30) smoking (41.2 ± 8.6% vs 53.3 ± 13.3%; P = 0.53) dyslipidemia (76.5 ± 7.4% vs 80 ± 10.7%; P = 0.82), diabetes (28.6 ± 7.7% vs 40 ± 13%; P = 0.51), stroke history (25.7 ± 7.5% vs 13.3 ± 9.1%; P = 0.46), myocardial infarction history (37.1 ± 8.3% vs 33.3% ± 12.6%; P = 0.79) or the use of anti platelet agent (63.3 ± 3.6% vs 59.2 ± 5.3%; P = 0.84). Only the initial value of TCPO2 was associated with critical limb ischemia (34 ± 14.2 mmHg, vs 52 ± 12.2 mmHg; P < 0.01).

#### Peripheral vascular state

During HD, the incidence of severe ischemia was statistically higher in limbs with pathologic ABI (ABI < 0.9 or ABI > 1.3) than in the normal ABI group, (80% vs 28.1% P = 0.0076) and (53.5% vs 28.1% P = 0.028). Critical ischemia occurred only in the two pathologic ABI groups (< 0.9, > 1.3, normal) in 8.3% and, 28% of cases, respectively.

Among limbs with a normal ABI, none had experienced a critical ischemia, and only 28% had a severe ischemia episode.

#### Arterial blood pressure (BP)

Systolic, diastolic and mean arterial pressure (MAP) trended to decrease during dialysis and reached a significant difference at 120 minutes compared to baseline values. At the end of dialysis, all parameters were similar to those at baseline. No difference was observed in blood pressure variation between the 3 ABI groups. Critical ischemia is related to a significantly larger decline in BP parameters: The critical ischemia group patient experienced a significantly larger decline in MAP (32.4 ± 26.1 mmHg vs 12.7 ± 10.7 mmHg; P = 0.007), in systolic BP (53.4 ± 50.7 mmHg vs 23.5 ± 20.9 mmHg; P = 0.005) and in diastolic BP (23.1 ± 14.9 mmHg vs 10.1 ± 8.7 mmHg; P = 0.001) than patients without ischemia.

#### Dialysis parameters

**Figure 3:**
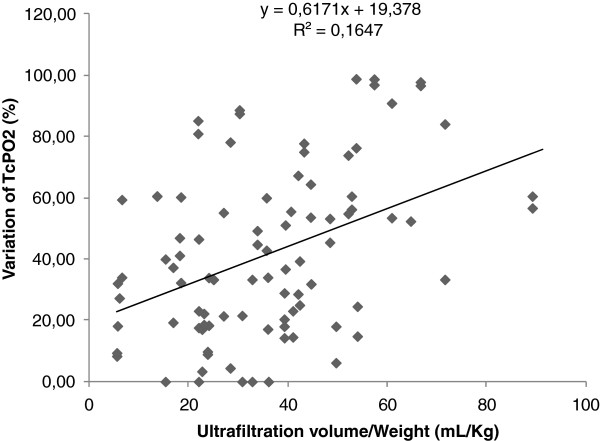
**Line graph shows the correlation between variations of TcPO2 and the value of the ultrafiltration/Weight in patients.** A positive relationship was observed (p < 0.001).

The mean total ultrafiltration volume in our study was 2459 ± 1197 ml. Adjusted to weight, the mean total UF/weight was 36.71 ± 18.05 ml/kg. A significant difference was observed in ultrafiltration variations between the group of patients whose limbs developed critical ischemia (45.55 ± 16.9 ml/kg vs 35.17 ± 18.2 ml/kg; P = 0.04) compared to the healthy group. This result was also present in case of severe ischemia (41.14 ± 20.15 ml/kg vs 31.28 ± 15.56 ml/kg; P = 0.03). A positive correlation was found between the variation of TCPO2 and the values of ultrafiltration/weight (rho 0.397; p = 0.001) (Figure 3). At the same time, mean decrease in RBV after 4 hours of dialysis was -7.4 ± 3.9%. However, this decrease was greater during the first two hours of the session (-5.4% ± 2.7%) compared to the last two hours (-2.0% ± 2%). There was no difference between groups regarding final RBV in cases of severe ischemia and critical ischemia.

### One year follow up

Thirteen patients (26%) died during the first year of follow-up; the main causes of death were infectious disease (n = 4) and cardiovascular complications (n = 5). Death occurred respectively in two third of cases in patients with ischemia (critical or severe). Six patients experienced new peripheral vascular events (revascularization and/or amputation). Five of them had an episode of critical ischemia during dialysis. When using combined criteria, including death from cardiovascular disease and vascular treatment, the frequency of pejorative outcome was five times higher in patients who had undergone at least one episode of critical ischemia during HD (55.7% vs 10.1% P = 0.01). In univariate analysis, age of patients, basal value of TCPO2, onset of critical ischemia and an ABI < 0.9 were more frequently observed in patients with pejorative outcome (*P* = 0.03, *P* = 0.048, *P* = 0.01 and *P* = 0.09 respectively).

## Discussion

This observational study investigated the effect of HD on the microcirculatory perfusion evaluated by TCPO2 measurements. The major findings are a decrease in TCPO2 during HD dependant on blood pressure reduction, ABI, level of ultrafiltration, as well as a high incidence of subclinical severe and critical limb ischemia during the session, which is independently correlated with a significant increase in pejorative outcome for patients.

Optimal fluid balance to maintain hemodynamic stability during hemodialysis procedure is essential to improve peripheral vascular perfusion. However, at the present time, hemodynamic stability is conventionally assessed on macrocirculation stability regarding systemic blood pressure and paying no attention to peripheral perfusion. Although ABI seems to be sufficient to predict poor outcome in haemodialysis patients, as confirmed by several previous studies. Nevertheless TCPO2, which is a real-time indicator of the perfusion of microcirculation can be a useful additional tool to alert the physician when distal injuries occur.

Mistrik et al. have recently demonstrated microcirculatory changes during HD by using laser Doppler methods [18]. Previously, Weiss et al. published results on the decrease in the TCPO2 measurement during hemodialysis and up to four hours after the end of the session [19]. Their results have been since confirmed in small cohorts without exact data on the influence of hemodialysis parameters on TCPO2 recording [14]. Our results are consistent with previous data but we have also investigated both the patient’s clinical parameters as well as some hemodialysis parameters to understand the mechanisms of microcirculatory injury. Peripheral vascular hemodynamic status is a condition which leads to the development of severe and critical ischemia during hemodialysis. While previous studies have failed to show a statistical impact on peripheral vascular status in the decrease in TCPO2, in this study we found, that the pathological vascular profile of limbs (PAD or mediacalcosis) revealed a major frequency of ischemia compared to the normal ABI group. Pathological limbs on the other hand had lower basal TCPO2 values and the same variation of arterial blood pressure compared to normal limbs. Adaptive autoregulation of distal flow seemed to be impaired when total volemia decreased. The increase in peripheral vascular resistance to maintain constant systemic arterial pressure lead to a drop in microcirculatory perfusion. Thus in patients with altered peripheral perfusion, hemodialysis would seem to be more deleterious.

A decrease in arterial blood pressure is one factor which is responsible for impairment in microcirculatory outflow. All patients experienced a significant decrease in arterial blood pressure during the hemodialysis session before recovering their basal values at the end of the session. The link between arterial systemic pressure and TCPO2 is well established. Megerman et al. have shown that TCPO2 decreased significantly and correlated positively with induced patient hypotension [20]. In the same way, tissue oxygen tension, measured at a variety of sites in both human and laboratory studies, appears to be a sensitive indicator of organ perfusion in different shock states [21,22]. Since intradialytic hypotension occurs in 25-55% of dialysis sessions [23], the concomitant decrease in TCPO2 in our study is not surprising [24-26]. However, in our study, the relative increase in TcPO2 and in MAP during the last hour of the session could be explained by effective vascular refilling of extra cellular volume. This adaptive mechanism may be connected to the profile of the variation of RBV as described by Santoro et al. [27]. Our results demonstrate a change in RBV in two phases, resulting in the occurrence of refilling during the second part of the session.

The other main parameter incriminated in distal microcirculatory injury is total ultrafiltration volume which is a predictive factor for the decrease in TCPO2 in our study. As the ratio between ultrafiltration and weight is considered as a better indicator of the intensity of volume loss, we choose to express our result using this ratio. However, results were similar when the total ultrafiltration was used (data not shown). It has been shown previously that microvascular perfusion is reduced by ultrafiltration and can be restored temporarily using the Trendelenburg position by increasing venous return or by administration of a small volume to replace the intravascular volume [28]. This adaptive procedure leads to an ischemia reperfusion phenomenon which is well known to aggravate tissue damage by producing reactive oxygen species (ROS) and inflammatory cytokines [29]. Stress and chronic inflammation are still the leading causes of atherosclerosis and cardiovascular mortality [30,31]. In our study, 45% of patients with normal basal distal perfusion experienced inadequate limb blood flow during HD subsequently developing microcirculatory injury leading to accelerated PAD. This result could explain that pejorative outcomes are five times higher in patients who had experienced limb ischemia episode during HD compared to other patients. Likewise, this phenomenon could explain in part why the rate of PAD procedures in HD patients compared to other chronic kidney disease patients is so high [32].

The major limitation of this study is the exclusion of patients with a recent cardiovascular event. It would have been interesting to analyse the results in those patients in whom peripheral vascular disease might be more prevalent. However, our cohort of patients already has a very high prevalence of cardiovascular comorbidities because of our recruitment in a tertiary center. Moreover, since our population is small, we decided to assess hemodynamic effects on the most stable population as possible to not over estimated the onset of vascular injuries and of pejorative outcomes. In addition, our cohort of patients already has a very high prevalence of cardiovascular comorbidities because of our recruitment in a tertiary center all patients received the same HD technique and it is impossible to generalize our results to others HD methods. Therefore, further studies are required to compare the impact of the different types of HD on microcirculatory perfusion. Additionally, since previous studies have shown that changes in TCPO2 may depend on type of dialyzer membranes [12,33] the exclusive use of high-flux synthetic membranes in our study may limit the generalization of our results. However, the trend is for maximum use of synthetic high flow membranes to reduce bio incompatibility and systemic consequences.

## Conclusion

In conclusion, our findings demonstrate that HD is responsible for microcirculatory injuries which are most important in patients with PAD. Better knowledge of the vascular status of the patient would seem to be necessary for patients undergoing HD. According to our results and previous reports, we believe that during HD, TCPO2 is useful for monitoring variations in distal perfusion and could assess tolerability against vascular damage. Therefore, changes in of session duration, in type of membranes used or in level of ultrafiltration could improve HD tolerance and probably overall patient prognosis.

## Competing interests

The authors declare that they have no competing interests.

## Authors’ contributions

YB had full access to all of the data in the study and takes responsibility for the integrity of the data and the accuracy of the data analysis. Study concept and design: YB, LB, SE. Acquisition of data: YB, LB, SE. Analysis and interpretation of data: YB, ND, SE. Drafting of the manuscript: YB, LB, ND, SE. Critical revision of the manuscript for important intellectual content: NC, CB, HL. All authors read and approved the final manuscript.

## Pre-publication history

The pre-publication history for this paper can be accessed here:

http://www.biomedcentral.com/1471-2369/15/4/prepub
